# Pressure Never Lies, but It Should Be Interpreted Thoughtfully: The Role of Hydrostatic Pressure in Coronary Physiology

**DOI:** 10.3390/jpm14030307

**Published:** 2024-03-14

**Authors:** Zsolt Kőszegi, Gábor Tamás Szabó

**Affiliations:** 1 Department of Cardiology, University of Debrecen, 4032 Debrecen, Hungary; nszgt@med.unideb.hu; 2 Kálmán Laki Doctoral School of Biomedical and Clinical Sciences, University of Debrecen, 4032 Debrecen, Hungary; 3 Szabolcs-Szatmár-Bereg County Hospitals and University Teaching Hospital, 4412 Nyíregyháza, Hungary; 4 Center for Biomedical Research and Translational Surgery, Medical University of Vienna, 1090 Vienna, Austria

## 1. Towards Personalized Medicine in Coronary Artery Disease

Personalized medicine shows promise for the management of patients with coronary artery disease (CAD). Tailored treatment strategies integrate precise physiological assessments of both the macro- and the microvasculature, making it possible to individually define disease characteristics. Consequently, effectively targeted interventional and pharmacotherapy can be applied to optimally treat patients while considering the specific requirements of the microvascular disease. Current guidelines reflect on this approach, emphasizing lesion-specific—i.e., personalized—decision making. Furthermore, in patients with anginal symptoms and without disease involving obstruction of the macrovasculature, the investigation of microvascular and coronary endothelial function should also be considered [1].

## 2. Invasive Assessment of Epicardial Lesions for Guiding Revascularization

Since the clinical introduction of coronary angioplasty in 1977, the need has arisen to determine the significance of coronary artery stenosis through intracoronary pressure measurement. Grüntzig acknowledged the potential of intracoronary pressure measurement in guiding coronary intervention and evaluating its outcome. However, technical limitations only allowed for the measurement of intracoronary pressure distal to the area of stenosis through the balloon catheter lumen.

In the 1990s, Nico Pijls and Bernard De Bruyne developed the concept of fractional flow reserve (FFR), which is based on the measurement of pressure drops caused by stenosis during hyperemia. Once the required technical tools became available, the use of this method became widespread.

Determining the functional significance of coronary stenoses using FFR has shown superiority compared to angiography only in clinical trials, which has led to its inclusion in the practical guidelines of coronary revascularization [2].

The concept of FFR is based on the principle that the pressure drop after stenosis is proportional to the decrease in perfusion pressure in the myocardium, and thus indicates the proportion of flow reduction compared to a non-stenotic artery during maximal vasodilation (hyperemia). Theoretically, this value can predict the degree of flow improvement after the complete removal of stenosis.

In the FAME-2 study, primary endpoints, including death, myocardial infarction and urgent revascularization, occurred more than twice as frequently after two years without revascularization below the threshold FFR value of 0.80 compared to the group which underwent intervention [3]. On the other hand, patients with FFR values above the threshold also showed significant risk for adverse cardiovascular events, with 9% experiencing unfavorable primary endpoints and 7.8% undergoing non-urgent revascularization as a secondary endpoint during the two-year follow-up. Conversely, among patients with reduced FFR (<0.80) but randomized to medical therapy only, 52.6% experienced no adverse events. Ultimately, the FFR threshold of 0.80 demonstrated a sensitivity of less than 50% in predicting the occurrence of any adverse event, highlighting the necessity for additional physiological prognostic indices in CAD.

## 3. The Concept of Flow Separation

FFR only reflects the hyperemic pressure gradient and cannot characterize resting flow condition, which can significantly influence the progression of epicardial plaque by exposing low and oscillating flow shear stress in the area of flow separation for much longer periods than even hyperemic flow. The flow separation index (FSi) is a recently introduced prognostic measurement that accounts for pathological turbulent flow [4]. According to this interpretation, the pressure–flow relationship derived from routine FFR measurement and three-dimensional (3D) parameters provides a novel index for the distinction between the resistance of laminar flow and the resistance of flow separation. According to the latter component, FSi was generated in the vessel-specific flow range, differentiating between benign laminar and pathologically disturbed (e.g., turbulent) flows. This concept is yet to be tested in clinical endpoint trials.

## 4. The Effect of Hydrostatic Pressure on Physiological Indices

Several consensus documents have been published addressing the proper execution of FFR measurement and thoroughly discussing the technical pitfalls that may arise during examination. However, these publications fail to adequately address that variations in hydrostatic pressure need to be considered during the measurement of resting or hyperemic distal pressure. A common example of the effect of hydrostatic pressure is when the distal pressure appears to be higher than the proximal pressure, leading a measured pressure ratio that appears to be greater than 1. This discrepancy can occur if the distal sensor is placed several centimeters below the level of the site of aortic pressure measurement at the tip of the catheter, such as the left circumflex artery, without significant stenosis.

Variations in hydrostatic pressure gradients have been shown to result in discernible differences in the ratio of resting distal coronary pressure to aortic pressure (Pd/Pa) and FFR values within specific coronary segments [5,6]. These differences are contingent upon the abovementioned vertical positioning of the vessel relative to the coronary orifice.

A recent meta-analysis provided compelling evidence on the clinical relevance of hydrostatic pressure by evaluating the functional results of stent implantation. FFR measured after stent implantation have been found to be different in the left anterior descending artery (LAD) and the non-LAD arteries, with an impact on prognostic value. These differences correspond to the hydrostatic effect on a vessel-specific basis [7].

It is increasingly acknowledged that hydrostatic pressure error needs to be considered when utilizing FFR for the purpose of making clinical decisions. It seems that an FFR threshold of 0.80 cannot be uniformly applied to all coronary lesions. Including the effect of hydrostatic pressure, the FFR threshold should identify functionally significant stenoses of <0.76 in the distal LAD, whereas values of >0.82 should be considered not significant in the circumflex artery.

## 5. Less Invasive Coronary Physiological Examinations: Virtual FFR

Despite the proven efficacy of invasive FFR measurement, it is not yet widely used in clinical practice due to its cost and technical complexity and a lack of awareness among healthcare providers. The development of examination procedures that can accurately and rapidly calculate FFR without expensive pressure wires may increase accessibility to treatment for a broader patient population [8].

Angiography-derived (or image-based) techniques can determine FFR through computational fluid dynamic (CFD) calculations or simplified fluid dynamic equations using 3D reconstruction from invasive angiography. Among the many software packages, including Cardiovascular Angiographic Analysis Systems for vessel Fractional Flow Reserve (CAAS vFFR; Pie Medical Imaging, Maastricht, The Netherlands), FFRangio (CathWorks, Newport Beach, CA, USA), caFFR (Rainmed, Suzhou, China), and Accu FFRangio (ArteryFlow Technology, Hangzhou, China), the most widely used software is Quantitative Flow Ratio (QFR) by Medis Medical Imaging (Leiden, The Netherlands), which has conducted the most clinical validation studies in CAD patients, including both chronic and acute coronary syndrome cases [9].

## 6. Invasive Microvascular Assessment

Coronary microcirculatory dysfunction (CMD) has been increasingly considered due to its role in the diagnosis and management of CAD. To detect CMD, coronary flow reserve (CFR) has become an established index, reflecting pathologies affecting both the epicardial arteries and microcirculation. The index of microcirculatory resistance (IMR) is a quantitative measure of resistance to blood flow in the coronary microcirculation, which reflects the status of the microvascular bed. Both indexes can be assessed through bolus thermodilution.

## 7. Angiography-Based Coronary Microvascular Assessment

Angiography-based methods calculate microvascular resistance by estimating coronary flow through angiographic frame counting and subsequently deriving distal coronary pressure using virtual FFR calculation. These techniques usually utilize the resting frame count to determine the mean transit time value corresponding to coronary flow and extrapolate the values to the hyperemic condition. These indices were compared with the reference method of invasive bolus thermodilution and were found to have good overall diagnostic accuracy in predicting abnormal invasive IMR.

A recent review highlighted that while angiography-based methods have good overall diagnostic performance, there are high limits of agreement between these methods and invasive IMR, as revealed by Bland–Altman analysis [10]. The authors pointed out the fundamental paradox of adenosine- and pressure-wire-free methods when microvascular resistance is calculated using assumed hyperemic coronary flow derived from average microvascular reactivity obtained from databases. Therefore, these methods could not reliably characterize individual microvascular function [10].

To overcome the problem of uncertain assumption, individual intracoronary pressure can be adopted for calculation following routine invasive FFR measurement. Tar et al. developed a method of calculating CFR and the resistive reserve ratio (RRR) using 3D coronary angiographic parameters and intracoronary pressure [11]. Here, microvascular resistance was calculated using coronary flow from invasive intracoronary pressure gradients measured during routine FFR investigations. This approach also incorporated individual variations in hydrostatic pressure, correcting the distal coronary pressure for hydrostatic pressure caused by the level difference between the tip of the catheter and the pressure wire sensor.

RRRp-3D demonstrated a good correlation with Doppler-derived RRR, with good limits of agreement with the Doppler-based method. These findings underscore the importance of accounting for hydrostatic pressure to avoid inaccuracies in calculating the driving pressure gradient [11].

## 8. Closing Thoughts

In this paper, we briefly summarize some important indices in coronary physiology and highlight one important and common challenge: when coronary pressure is precisely measured, it never lies, but it is also affected by hydrostatic pressure. Therefore, hydrostatic pressure should be considered when calculating physiological indices.
